# Augmented Reality in Radiography Education: Opportunities, Limitations and Lessons From Virtual Reality Research

**DOI:** 10.1002/jmrs.70038

**Published:** 2025-10-28

**Authors:** James Hayes

**Affiliations:** ^1^ Virtual Medical Coaching Christchurch New Zealand

## Abstract

Augmented reality (AR) is gaining traction in radiography education, offering tactile engagement enhanced by digital overlays. This editorial compares AR with virtual reality (VR), highlighting both hidden and visible costs, and draws lessons from VR research to guide future adoption. It calls for direct comparative studies to inform evidence‐based implementation.

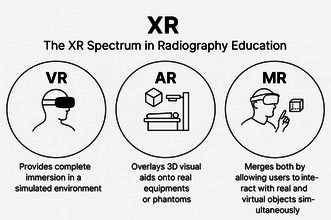

In radiography education, extended reality (XR) is the combined spectrum of immersive technologies that include virtual reality (VR), augmented reality (AR) and mixed reality (MR). VR immerses the user in a complete digital world, while AR imposes virtual entities over the real world, such as the AR position tool assessed by Nagamata et al. [1]. MR blends the two methods and allows for interaction between virtual and real entities in real time. This editorial has particular emphasis on AR and VR because these modalities already have the broadest evidence base established in radiography education.

Figure 1 offers a visual summary of XR technologies. VR provides complete immersion in a simulated environment, AR overlays 3D visual aids onto real equipment or phantoms, and MR merges both by allowing users to interact with real and virtual objects simultaneously.

**FIGURE 1:**
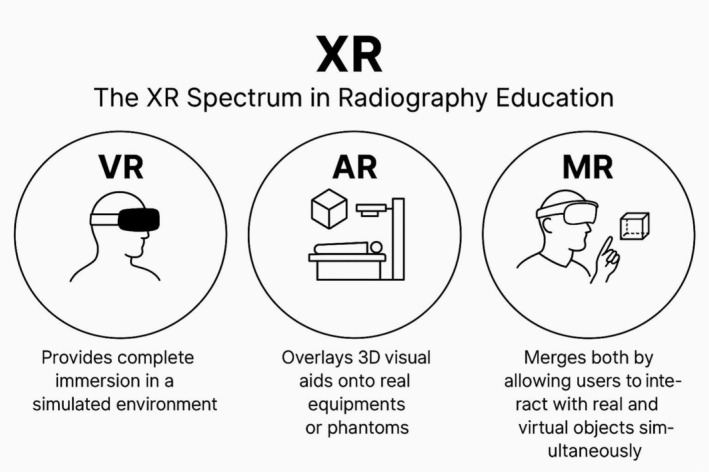
The XR spectrum in radiography education. AR, augmented reality; MR, mixed reality; XR, extended reality.

Nagamata et al. [1] discuss the educative potential of AR as a teaching medium for radiographic positioning using an open‐source platform. Their study indicates that AR can encourage students, give them vision‐based instant feedback, and enable accurate positioning during projection radiography. This is a timely contribution, as medical imaging education is increasingly investigating XR technologies to enhance spatial awareness and procedural skills.

A strength of AR is that it directly augments physical‐world practice. Tactile interaction with phantoms or live subjects is preserved, while digital overlays provide additional guidance. This combination may support kinaesthetic learning more effectively than digital‐only tools. At the same time, AR implementation requires attention to cost and sustainability. Although Nagamata et al. used open source software, staff time for software engineering, 3D modelling, testing and course integration is a material cost. Most current AR deployments rely on printed visual markers for reliable registration, and many are bespoke builds maintained in‐house, which concentrates engineering effort within the institution. For universities with strong in‐house development teams, this model may be sustainable and scalable to large cohorts without per‐user licencing fees (Dr. Patricia C. Ramos, Medical Director, Centre for Innovative Medical Simulation, personal communication, 5 September 2025).

The perception of AR as an inexpensive substitute for VR must be moderated. Both approaches carry costs, with AR requiring staff development and maintenance, and VR involving licencing. Their strength lies less in relative cost than in aligning each technology with institutional resources and learning objectives. AR excels when experiential engagement is the priority, whereas VR enables completely reproducible, immersive scenarios. Future studies that directly compare AR and VR, evaluating both educational effectiveness and long‐term ownership costs, will better inform educators' decisions grounded in pedagogy, resources and scalability.

VR provides a useful point of comparison because it has been in use longer and has accumulated a broader evidence base. VR platforms now operate on untethered headsets or standard PCs, require only small teaching spaces, and integrate with learning management systems (LMS) and objective structured clinical examination (OSCE) workflows. Studies have shown VR to improve positioning accuracy in projection radiography [2], enhance dose awareness and protective behaviours in cardiac catheterisation [3, 4, 5] and strengthen the confidence, adaptability, and clinical readiness of newly qualified radiographers [6]. These findings illustrate what is achievable when immersive tools are integrated into curricula.

AR provides a vocational bridge between the classic simulation and the complete immersion of the fully virtual. Its ability to integrate digital overlays with physical interaction facilitates the building of skills that are enhanced by being supplementary to, not competitive with, VR. As Nagamata et al. [1] have demonstrated, long‐term usefulness relies on sustained integration, scrutiny, and congruence with the goals of the curriculum. Both AR and VR together constitute a continuum of media by which radiography educators may optimise learning outcomes and learning experience.

## Conflicts of Interest

James Hayes is the founder and a director of Virtual Medical Coaching Ltd., a company that develops and licences immersive XR training software for allied health education, including radiography and radiation safety. He is currently completing a Postgraduate Diploma in Computer Science, specialising in Artificial Intelligence, at Stanford University. No external party influenced the writing of this piece, and no third‐party funding was received for it.

## Linked Articles

This article is linked to Nagamata papers. To view this article, visit https://onlinelibrary.wiley.com/doi/10.1002/jmrs.70019

## Data Availability

The data that support the findings of this study are available from the corresponding author upon reasonable request.

## References

[1] M. Nagamata , “Augmented Reality Application for Radiographic Positioning Training in Undergraduate Radiography Students,” Journal of Medical Radiation Sciences 72 (2025): 512–518, 10.1002/jmrs.70019. PMC12661066 40847530

[2] S. Rowe , A. Garcia , and B. Rossi , “Comparison of Virtual Reality and Physical Simulation Training in First‐Year Radiography Students,” Journal of Medical Radiation Sciences 70 (2023): 155–164, 10.1002/jmrs.616. PMC10258633 36502536

[3] M. Fujiwara , S. Fujimoto , R. Ishikawa , and A. Tanaka , “Virtual Reality Training for Radiation Safety in Cardiac Catheterisation Laboratories – An Integrated Study,” Radiation Protection Dosimetry 198, no. 3 (2024): 276–287, 10.1093/rpd/ncae187. PMC11413571 39244378

[4] W. Mwangi and Y. Tanaka , “Comparative Effectiveness of Immersive Virtual Reality and Traditional Didactic Training on Radiation Safety in Medical Professionals: a Crossover Study,” Journal of Medical Radiation Sciences 72 (2025): S52–S60, 10.1002/jmrs.867. 40033760 PMC12449611

[5] K. K. Khamis , A. S. Bello , and M. L. Abdullahi .“Assessing the impact of virtual reality training on radiation dose reduction among interventional radiology nurses: a multicenter crossover study,” Journal of Radiology Nursing 44, no. 3 (2025): 300–305, 10.1016/j.jradnu.2025.05.005.

[6] H. Karimi , S. Clarke , and E. Watson , “Comparing Clinical Preparedness of Newly Qualified Diagnostic Radiographers Trained With Immersive Virtual Reality vs Traditional Simulation: A Mixed‐Methods Study,” Journal of Medical Radiation Sciences 72 (2025): S70–S78, 10.1002/jmrs.882. 40325902 PMC12449592

